# Impact of Online Knowledge and Skills Learning on Millennial Learners Within Emergency Medicine: A Retrospective Data Review

**DOI:** 10.7759/cureus.20626

**Published:** 2021-12-22

**Authors:** Khalid Bashir, Shahzad Anjum, Mohamed Dewji, Zeenat Khuda Bakhsh, Hamza Said Wali, Aftab Azad

**Affiliations:** 1 Medicine, Qatar University, Doha, QAT; 2 Emergency Medicine, Hamad Medical Corporation, Doha, QAT; 3 Medical Education, Primary Health Care Corporation, Doha, QAT; 4 Emergency Medicine, Qatar University, Doha, QAT

**Keywords:** online teaching method, millennial learner, distant education, skilled learning, online medical education

## Abstract

Background: Emergency Medicine didactic teaching has traditionally been delivered through face-to-face (F2F) lectures. However, during the pandemic of COVID-19, the didactic teaching was switched to online through using Microsoft Teams. The aim of this study was to assess the impact of online learning in the knowledge and skills acquisition of millennial learners based within emergency medicine.

Methodology: This was a retrospective review of assessment data. Over a period of 10 months (August 2019 to June 2020), each resident was exposed to traditional F2F teaching for a period of four months and then online teaching in a crossover manner. After each method, there were two types of assessments, multiple-choice questions (MCQs) and computer-based objective structured clinical examination (OSCE). A total of 20 MCQs with one correct answer, totaling 20 marks, and 20 OSCEs consisting of an image or a video with five options, each option carrying one mark, totaling 100 marks were used at each assessment point. A student t-test was used to compare the two groups of results.

Results: The total number of participants was 49 (n=49). All residents belonged to the millennial generation. Fourteen were female and 35 were male. The mean MCQ 1 score after F2F teaching was 12.16 (SD=1.688), whilst the mean MCQ 2 score after online teaching was 13.40 (SD=1.861). The mean computer-based OSCE 1 score after F2F teaching was 64.45 (SD=5.895), whilst the mean OSCE 2 score after online teaching was 65.57 (SD=5.969). Ten out of 49 students (20.4%) failed the MCQ exam after F2F teaching, whilst 6/49 students (12.2%) failed the MCQ test after online teaching. Seven out of 49 students (14.3%) failed the OSCE exam after F2F teaching, while six out of 49 students (12.2%) failed the OSCE exam after online teaching. There was a statistically significant improvement in the MCQ score after online teaching as compared to F2F teaching (P-value 0.0003), whilst there was no statistically significant change in the OSCE between the two-teaching methods (P-value 0.3513).

Conclusion: Both F2F and online teaching methods resulted in a significant improvement in the knowledge and skills of emergency medicine residents. Online education resulted in a statistically significant improvement of MCQ score as compared to F2F teaching. The difference in MCQ score may be due to millennial learners, who traditionally benefit proportionately more from self-learning that is primarily online.

## Introduction

Online learning is a term that describes a learning process that uses the internet and other technological tools to impart knowledge and skills outside of a conventional classroom. This platform of learning is in contrast to the traditional face-to-face (F2F) learning process that takes place within the confines of a classroom. Medical institutions around the world have utilized various innovative and creative strategies to teach and train their students especially at the emergence of the COVID-19 pandemic, using available applications and software such as Google Classroom, Zoom, and Microsoft Teams to deliver courses online. To ensure not only completion of a course and delivery of lectures but also to stay in constant contact with the learners, online learning through virtual classes was initiated to grow the certainty and confidence of the students within their Faculty even as the world was battling the COVID-19 pandemic [1]. Though there are obvious advantages of online learning, some experts and researchers in education have seriously advised against replacing the traditional classroom F2F learning with online learning platforms. However, a majority of these experts and researchers suggest that online learning can be used to support classroom F2F [2]. A systemic review evaluating the impact of online or “blended learning vs F2F learning of clinical skills in undergraduate nursing students” has shown that online learning for teaching clinical skills is no less effective than traditional means [2].

Emergency medicine specialists are trained to handle unscheduled and undifferentiated patients of all ages. They serve as frontline workers, in coordination with Emergency Medical Services and are primarily responsible for initiating resuscitation, stabilization, and performing the germinal investigations and interventions necessary for the diagnosis and treatment of illnesses or injuries within the acute phase. This specialty requires adequate knowledge and skills in the prevention, diagnosis, and management of acute and urgent aspects of illness and injury affecting patients belonging to all age groups. The scope of this aspect of medicine also entails an understanding of the development of pre-hospital and in-hospital emergency medical systems and the skills to support this.

Millennials, also called Generation Y or Gen Y, are the demographic cohort following Generation X and preceding Generation Z. Researchers and popular media classify those born between the early 1980s and mid-1990s to early 2000s as Millennials, with the generation typically being defined as people born from 1981 to 1996 [3]. Millennials are known as the first global generation that grew up in the internet age. The Millennials generation is commonly marked by higher usage of and knowledge of the internet, mobile devices, and social media. Hence, they are sometimes termed digital natives [4]. The millennial learner is a term used to describe students belonging to the millennial demography. This set of learners relies majorly on technology and the internet to facilitate their learning process. They appreciate virtual means of learning more than the generation before them. Their congenial relationship with technology and virtual environments cannot be overlooked when we think of the ways to help millennials learn better.

The World Health Organization (WHO) in January 2020 announced COVID-19, a new coronavirus disease outbreak and was later declared as a pandemic [5]. This disease has caused significant disruption and havoc across the globe and it is a public health crisis of worldwide importance. The emergence of the COVID-19 pandemic forced the world into creating alternative means of carrying out activities that normally would require contacts between individuals or bring persons into close proximity. The use of these alternative means became an absolute necessity when the World Health Organization (WHO) recommended social distancing as a preventative measure against the spread of COVID-19 and strictly warned against any activity that would bring people into close proximity with each other. The education sector was not spared from the effect of the pandemic, schools were shut down and the traditional F2F learning process within the confines of a classroom was brought to an abrupt pause (and effectively a halt) across the globe. This shutdown stimulated the growth of online educational activities in order to avoid interruption within education. This is because the imparting of knowledge and skills cannot be impeded in life wherever possible, for a continuation of any and all successful development. Other learning platforms outside of the conventional classroom were adopted by many in the education sector and online learning became the “new normal” within the education systems of most countries. A majority of faculties have been involved in how best to offer course materials, involve students, and perform evaluations online. However, some experts and researchers in education still advocate for the return of students to the conventional classrooms, as they believe that long-term use of online platforms for learning and keeping students outside of the conventional classroom will have a detrimental effect on the education system. Millennial learners are more comfortable with this new way of learning than the generation [3].

The imparting of knowledge and skills in emergency medicine has always been done through the traditional F2F method of learning and due to its wide scope and the technicalities involved, experts in this field believe it must remain that way, but the emergence of COVID-19 has forced even the stakeholders in emergency medicine to accept other learning platforms outside of the classroom and embrace online learning. This study is designed to assess the impact of online learning on the level of knowledge and skills of emergency medicine millennial residents and review if this has consequently improved, stayed stable, or deteriorated their performance within two different forms of assessments.

## Materials and methods

The emergency medicine residency training program (EMRTP) runs for four years and had 49 residences at the time of the study, including rotating residents from other specialties. The EMRTP has been recognized by the Accreditation Council of Graduate Medical Education-international (ACGEM-I). Every year, the residency program starts on July 1. The first formative assessment was done in December 2019. In February 2020, the education went online to comply with the national pandemic guidelines. The second formative assessment was completed in July 2020. The comparison between the two assessments was done by using the student's t-test. The multiple-choice question (MCQ) exam consisted of 20 questions with one correct answer. The MCQs were prepared by the EMRTP program director (PD) from the curriculum blueprint. Each MCQ carried one mark (Tabel 1). There were a total of 20 marks for the MCQ.

**Table 1:** Multiple-choice question (MCQ) from from the curriculum blueprint

Options	Yes	No
A. 1 mg		
B. 10 mg		
C. 100 mg		
D. 200 mg		
E. 300 mg		

**Figure 1:**
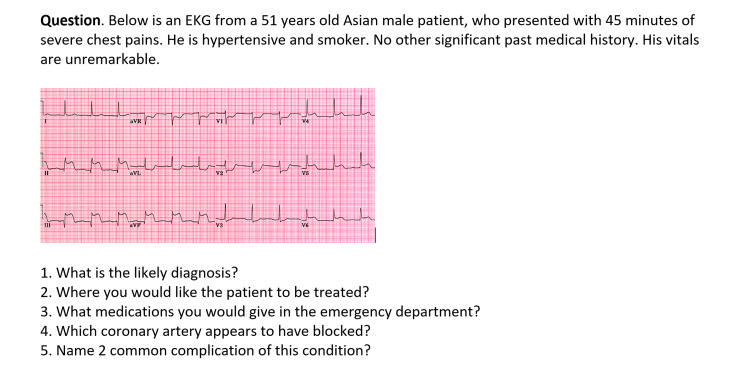
Computerized OSCE OSCE - objective structured clinical examination

The computer-based objective structured clinical examination (OSCE) question consisted of a clinical scenario, an image, or video followed by five questions (Figure 1), each question carried one mark. There were a total of 20 OSCE questions carrying 100 marks. The MCQs included different areas of the curriculum taught during spring and autumn. The residents undergo formative assessments at the end of each month that includes MCQs and OSCE. The residents have access to emergency medicine books electronically provided to the hospital such as Tintinalli, and Rosen’s Emergency Medicine. All residents were included there were no exclusion criteria. The test was administered online, and the residents answered the questions on the paper, they then compared their answers with the answers provided by the program director. Each resident then emailed his score to the PD. MCQ was allocated 30 minutes to complete while OSCE took 45 minutes to complete.

The retrospective data were collected in Microsoft XL Microsoft Corporation Redmond WA. The data were then transferred to the statistical package “Stata” (version 15MP, StataCorp, College Station, TX, USA). The data were normally distributed and presented as mean and standard deviation. Statistical significant parameters were set at P 0.05 and the confidence interval was reported at the 95% level. A student t-test was used to compare the two groups.

## Results

**Figure 2:**
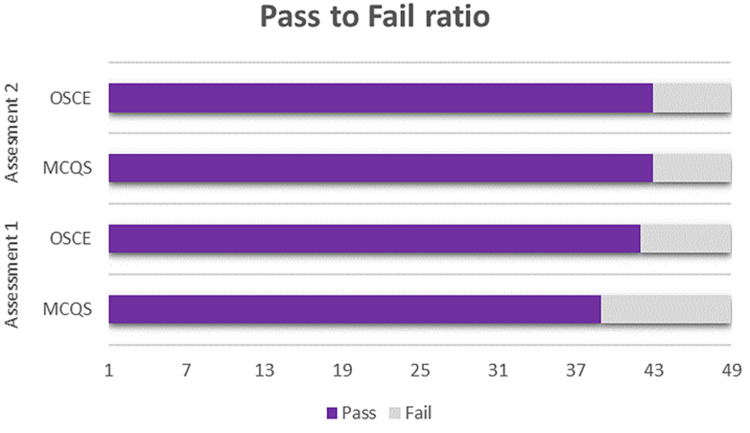
Pass-to-fail ratio in both assessments OSCE - objective structured clinical examination, MCQS - multiple-choice questions

**Figure 3:**
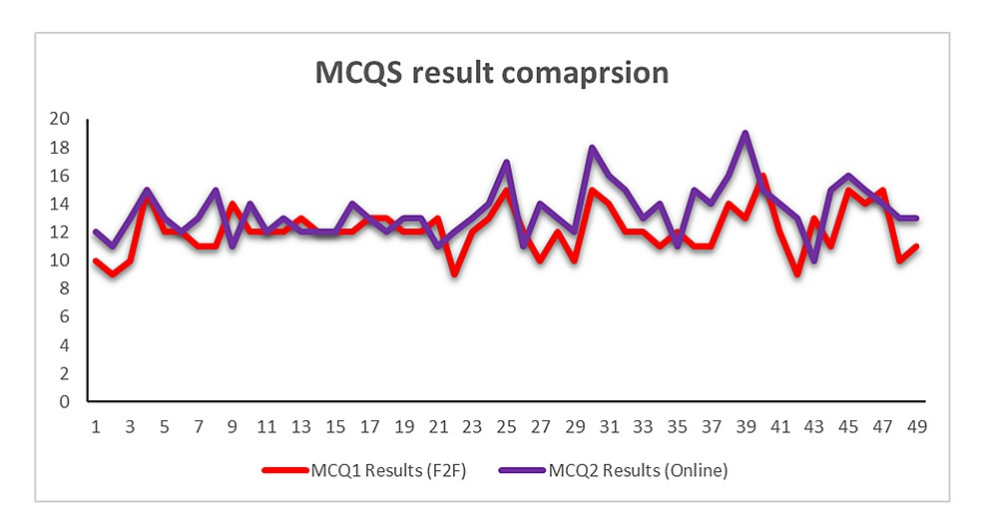
Face-to-face vs online MCQs comparison result MCQs - multiple-choice questions

**Figure 4:**
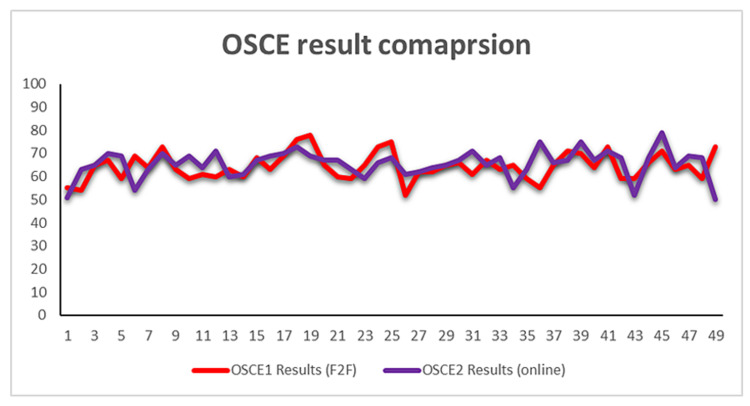
Face-to-face vs online OSCE comparison result OSCE - objective structured clinical examination

A total of 49 residents’ (14 female and 35 male) data were collected (Table 2). Ten out of the 49 residents failed the first MCQ (MCQ 1) test after F2F teaching. This represents a 20.4% failure rate, while six out of the 49 (12.2%) emergency medicine residents failed the second MCQ test (MCQ 2), which followed the online teaching (Figures 2, 3). Seven out of the 49 (14.3%) millennial learners failed the first computer-based OSCE test (OSCE 1) after F2F teaching, while six of the 49 (12.2%) learners failed the OSCE 2, which followed the online teaching (Figure 4). The study showed a statistically significant (P-value 0.0003) improvement in the MCQ score after online teaching as compared to F2F teaching, though there was no statistically significant improvement in the OSCE outcomes between the two-teaching method (P-value 0.3513).

**Table 2:** Male and female participants

Gender		Frequency	Percent	Valid Percent	Cumulative Percent
Valid	F	14	28.6	28.6	28.6
	M	35	71.4	71.4	100.0
	Total	49	100.0	100.0	

## Discussion

The study looked at knowledge and skills assessment outcomes through MCQ and computer-based OSCE after two different educational approaches, F2F and online. Both educational approaches resulted in improving the knowledge and skills of residence. The improvement in the MCQ test was statistically better after online learning. However, there was no statistical difference in the improved outcomes of knowledge in computer-based OSCE tests between the two educational approaches.

The emergence of COVID-19 has caused a wide acceptance of online learning by the various stakeholders within education as against F2F learning. This acceptance is due to the fact that social distancing serves as one of the major preventing measures against COVID-19 and though learning must continue it cannot be done in close proximity. Hence, the adoption of online learning as means of imparting knowledge and skills during the pandemic. This study was designed to assess the impact of online learning on emergency medicine millennial learners.

This study revealed that online learning was more effective in improving the knowledge and skills of emergency medicine millennial learners than traditional F2F learning as measured by MCQs, and thus, suggesting online learning as a better tool in imparting knowledge to millennial learners in emergency medicine. The result from this study agrees with other research on millennial learners which showed that they learn more and appreciate knowledge and skills passed across to them using the internet and other technological tools. Oomen-Early and Early in their report in 2015 stated that most millennial learners entering college in this present day, do not know a world without computers or new media. Hence, one of the biggest challenges facing higher education today is bridging the gap between these “digital natives” and faculty who may see themselves as digital immigrants. According to their report, some scholars believe that higher education faculty, in general, are using 20th-century methods to teach 21st-century students [6]. This gap could be the reason for the results recorded within this study and may be responsible for the higher performance in millennial learners after online learning than the F2F learning.

Though the study suggests online learning as a better tool in improving knowledge and skills in emergency medicine millennial learners, it is relevant to be aware of a paradox (digital natives lacking digital literacy) that exist among Millennials. It is accepted that even though some millennial learners may be immersed in new media and technologies, they may not fully understand how to harness this technology to benefit their learning [7]. Many researchers have in their reports described Millennials as having an innate flair for using technology to achieve their goals. However, some empirical studies such as that of Somyürek and Coskun [8] contradict this claim; they reported that some Millennials rarely use emerging technologies to retrieve, assess, store, produce, present, and exchange information, all of which are basic skills of digital competency. Hence, it is imperative that a comprehensive learning method be designed to accommodate millennial learners even after the pandemic.

Several limitations need to be considered in relation to this study. First, the study looked at the results of the MCQ exam and computerized OSCE which may not represent the full effort by the residents. The residents may have spent extra time spent online resulting in better MCQ results. A focus group could have been a better approach to capture this information. The focus group was not possible, as this was a retrospective review of the data, and was difficult to interview residents during the height of the pandemic. Second, this was a single hospital study and the results may not be generalizable to other hospitals. Finally, we have only used MCQs and OCCE tests for the assessment; it would have been better to use short answer questions and course work as recommended blooms taxonomy [9]. Due to the acute nature of the pandemic and the availability of retrospective data, this was not possible, both for the residents and the faculty. Finally, we presumed that residents have been honest in reporting their scores. It would have been better to examine them on-site along with invigilators, unfortunately, due to pandemic regulation, this was not possible.

## Conclusions

The study has compared the results of MCQs and OSCE after F2F and online educational methods. The traditional F2F and online teaching approach resulted in a significant improvement in the knowledge and skills of emergency medicine residents. There was a statistically significant improvement in the MCQ score following online education as compared to F2F teaching. The difference in MCQ score may be due to Millennial learners, who traditionally benefit more from online-based self-learning.
